# Cell Free Expression of hif1α and p21 in Maternal Peripheral Blood as a Marker for Preeclampsia and Fetal Growth Restriction

**DOI:** 10.1371/journal.pone.0037273

**Published:** 2012-05-16

**Authors:** Osnat Ashur-Fabian, Gil M. Yerushalmi, Shali Mazaki-Tovi, David M. Steinberg, Inbal Goldshtein, Michal Yackobovitch-Gavan, Eyal Schiff, Ninette Amariglio, Gideon Rechavi

**Affiliations:** 1 Cancer Research Center, Chaim Sheba Medical Center, Tel Hashomer, Israel; 2 Department of Human Molecular Genetics and Biochemistry, Sackler School of Medicine, Tel-Aviv University, Tel Aviv, Israel; 3 Department of Obstetrics and Gynecology, Chaim Sheba Medical Center, Sackler School of Medicine, Tel-Aviv University, Tel-Hashomer, Israel; 4 Department of Statistics and Operations Research, Tel Aviv University, Tel Aviv, Israel; Virginia Commonwealth University, United States of America

## Abstract

Preeclampsia, a severe unpredictable complication of pregnancy, occurs in 6% of pregnancies, usually in the second or third trimester. The specific etiology of preeclampsia remains unclear, although the pathophysiological hallmark of this condition appears to be an inadequate blood supply to the placenta. As a result of the impaired placental blood flow, intrauterine growth restriction (IUGR) and consequential fetal oxidative stress may occur. Consistent with this view, pregnancies complicated by preeclampsia and IUGR are characterized by up-regulation of key transcriptional regulators of the hypoxic response including, hif1α and as well as p53 and its target genes. Recently, the presence of circulating cell-free fetal RNA has been documented in maternal plasma. We speculated that pregnancies complicated by preeclampsia and IUGR, will be associated with an abnormal expression of p53 and/or hif1α related genes in the maternal plasma. Maternal plasma from 113 singleton pregnancies (72 normal and 41 complicated pregnancies) and 19 twins (9 normal and 10 complicated pregnancies) were collected and cell free RNA was extracted. The expression of 18 genes was measured by one step real-time RT-PCR and was analyzed for prevalence of positive/negative expression levels. Results indicate that, among the genes examined, cell free plasma expressions of p21 and hif1α were more prevalent in pregnancies complicated by hypoxia and/or IUGR (p<0.001). To conclude, we present in this manuscript data to support the association between two possible surrogate markers of hypoxia and common complications of pregnancy. More work is needed in order to implement these findings in clinical practice.

## Introduction

Preeclampsia occurs in 6% of pregnancies and is one of the most common, dangerous, unpredictable complications of pregnancy [1]. The cause of preeclampsia remains unclear, although the pathophysiology appears to be inadequate blood supply to the placenta leading to hypoxic environment [2], [3], [4] which may lead to fetal growth restriction. Intrauterine growth restriction (IUGR), even in the absence of preeclampsia, is a challenging obstetric complication with increased rate of fetal and perinatal morbidity and mortality [2], [4], [5].

Oxygen deprivation leads to the up-regulation of genes mainly by the hypoxia-inducible factors (HIFs) [6], [7]. Hif1α is a major regulator of cellular and systemic responses to hypoxia [8], [9], [10], [11], [12], [13]. In addition, hif1α regulates TGFβ3, a target gene induced by hypoxia both *in vivo* and *in vitro* in trophoblast cells [14], [15], [16], [17]. The two genes, hif1α and TGFβ3 are overexpressed in pregnancies complicated by preeclampsia and IUGR [15], [18], [19], [20], [21].

p53 is a central tumor suppressor gene and a major transcriptional activator of a spectrum of genes under hypoxic conditions [22], [23], [24], [25], [26]. In placentas of pregnancies complicated by fetal growth retardation, enhanced apoptosis and up-regulation of p53 was reported [27].

A few years ago cell-free fetal RNA was discovered in the maternal plasma [28], [29], enabling non invasive measurements of placental/fetal gene expressions [30], [31]. Given the limitations of the current modalities, there is an urgent need for the development of a more refined and reliable approach for fetal stress/growth monitoring. We speculated that abnormal gene expression of p53 related genes and/or hif1α related genes may be more prevalent with preeclampsia complicated pregnancies as well as IUGR. Following evaluation of the expression of 18 different genes in the maternal plasma we discovered candidate biomarkers for the identification of complicated pregnancies and fetal growth restriction.

## Materials and Methods

### Subject recruitment

Approval to undertake the study was granted by the institutional human research and ethics committee. Written, informed consent was obtained prior to subject assessment. Women were recruited between March 2007 and September 2008 from the Obstetrics and Gynecology department at Chaim Sheba Medical Center, Israel. A total of 132 blood samples were collected (**Table S1**), 113 from singleton pregnancies (72 healthy pregnant women and 41 women with complicated pregnancies) and 19 twins (9 normal twin pregnancies and 10 complicated twin pregnancies). The selection criterion for complicated pregnancies was IUGR with/without preeclampsia. IUGR was defined as birth weight below 10th centile [2], [5] of customized birth weight adjusted for singleton or multiple gestation, sex of baby and gestational age, developed for local Israeli subjects [32]. Preeclampsia was defined as previously described [33]. Complicated pregnancies were designated as hypoxic pregnancies (H) whereas other pregnancies were designated as normal (N). Multiple parameters were collected for each case including: Maternal age, weight, parity, medications, smoking status, maternal hemoglobin concentration, fetoplacental Doppler studies, newborn estimated and actual birth weight, 1 and 5 minutes Apgar scores.

#### Blood collection

15 ml blood samples were collected from all participants. Samples were prepared as was previously described by Ng et al [28]. The blood samples were collected in EDTA-containing tubes centrifuged at 1,600× *g* for 10 minutes at 4°C (to remove nucleated cells from the blood sample). Plasma was then carefully transferred into 1.5 ml eppendorf tubes. The plasma samples were re-centrifuged at 16,000× *g* for 10 minutes at 4°C and the supernatants were collected into fresh polypropylene tubes.

#### Target selection

The basis for selection of candidate biological markers was genes associated with hypoxia (**Table S2**). **Table S2A** depicts three different target genes including: hif1α, a key transcriptional regulator of the hypoxic response and it's downstream activated genes, VEGF-A (VEGF) and TGFβ3 which were shown to be over-expressed in preeclampsia [18], [20], [21]. **Table S2B** depicts p53 and fourteen downstream p53 target genes, which were shown to be elevated by more than 10-fold following p53 activation based on previous microarray analyses [23], [24].

#### RNA Extraction

1.6 ml plasma was mixed with 2 ml TRIzol LS reagent (Invitrogen, Carlsbad, CA) and 0.4 ml chloroform as previously described [34]. The mixture was centrifuged at 11,900× *g* for 15 minutes at 4°C and the aqueous layer was transferred into new tubes. One volume of 70% ethanol was added to one volume of the aqueous layer and transferred to an RNeasy minicolumn (RNeasy mini kit, Qiagen, Valencia, CA) following the manufacturer's recommendations. On-column DNase treatment was carried out to remove any contaminating DNA (RNase-Free DNase Set, Qiagen, Valencia, CA). Total RNA was eluted in 30 µl RNase-free water and stored at −80°C. RNA was extracted from each sample 2–3 independent times and was assessed for quality by measuring actin beta expression (assay number Hs99999903_m1, Applied Biosystems, Foster City, CA) using a one-step real-time quantitative reverse transcription (RT) PCR as detailed below.

#### Real-Time Quantitative RT-PCR

Amplification of specific cell free mRNA of the 18 genes selected for analysis was conducted using a one-step real-time quantitative RT-PCR with specific primers and probe for each gene of interest. One step RT-PCRs were set up according to the manufacturer's instructions (EZ rTth RNA PCR reagent set, Applied Biosystems, Foster City, CA) in a reaction volume of 20 µl. In detail, 5 µl extracted plasma RNA was amplified using various Taqman assays consisting of appropriate probe and PCR primers (**Table S2A–B**, Applied Biosystems, Foster City, CA). The RT-PCR thermal profile used for all primers designed was as follows: The reaction was initiated at 50°C for 2 minutes to allow the uracil N-glycosylase to become active, followed by reverse transcription at 60°C for 30 minutes and a 5 minute denaturation at 95°C. Next, 50 cycles of PCR were carried out as follows: 20 seconds of denaturation at 94°C followed by 1 minute of annealing/extension at 60°C. Each plasma RNA sample was analyzed for the expression of the 18 selected genes in duplicates, from at least two independent RNA extractions. To correct for differences in both RNA quality and quantity between samples, data were normalized using the Delta Ct method in which the ratio of the target gene cycle threshold (Ct) to the Ct of actin beta was calculated for each sample (**Table S3A**). As all 18 genes, including the normalizator actin beta, were measured simultaneously in the same assay for each RNA sample, relative quantification and not absolute quantification approach was utilized. Following normalization of each sample for actin beta (Delta Ct), the comparative threshold cycle (2∧ Delta-Delta Ct) method was used to calculate the fold-change of the different genes relative to a calibrator (**Table S3B**). RNA extracted from human leukemia cell line, k-562 cells (ATCC CCL-243), which expresses all target genes, served repeatedly as a positive control (Delta-Ct values (average±STDEV) are presented in the bottom of **Table S3**) and a calibrator in each and every assay (i.e., calibrators are assigned a value of 1 by definition). No template controls (NTC) were also included in every analysis for each gene.

#### Statistical analysis

Hypoxic pregnancies were compared to normal pregnancies using chi-square tests or Fisher's exact test for categorical variables, t-tests for numerical variables with normal distributions and the Wilcoxon test for numerical variables with non-normal distributions. Multivariate logistic regression was used to study the joint effects of variables in distinguishing between hypoxic and normal pregnancies. Possible relationships of numerical variables to genetic markers were explored using analysis of covariance (ANCOVA), adjusting for the type of pregnancy (hypoxic or normal). For outcomes that did not meet the ANCOVA assumptions (normal distribution and constant error variance), p-values were determined by the bootstrap. In all analyses, p-values<0.05 were considered statistically significant.

## Results

### Characteristics of the hypoxic and control groups

132 samples were included in the study cohort (**Table S1**). 19 twin pregnancies were evaluated by a separate analysis. A total of 113 blood samples from pregnant women, 72 samples from healthy pregnancies (marked as normal, N) and 41 from complicated pregnancies (marked as hypoxic, H) were analyzed (**Table S4**). There was no age difference between the groups (p = 0.197). Birth week, median adjusted fetal weight (centile) and median adjusted birth weight were, as expected, significantly lower in the hypoxic group (p<0.001).

### High frequency of p21 and hif1α positive expressions in hypoxic versus normal pregnancies

Eighteen genes were selected for analysis in this study (**Table S2**), with 13 genes not expressed in all samples. Transcripts of 5 genes including p21, hif1α, mdm2, VEGF and ERCC5 were detected in the cell free maternal plasma samples and were chosen for further assessment. The relative expression level of p21, hif1α, mdm2, VEGF and ERCC5 genes, as calculated by the Delta-Delta Ct method (**Table S3B**), was similar between normal and complicated pregnancies (p>0.05), however there was a clear difference in the frequency of positive/negative expressions of several genes between the two groups. As the aim of this study was to discover a possible marker that will differentiate best between complicated and normal pregnancies, we were interested in identifying genes that are expressed with higher incidence in complicated pregnancies. We therefore focused on analyzing the differences between the two groups by a categorical positive (marked by +) or negative (marked by −) gene expression (**Table S1**).

Chi square (χ^2^) tests were carried out to compare the prevalence of expression of these genes in the hypoxic and control groups (**Table 1**). Significantly higher frequency of positive p21 and hif1α expression was observed in hypoxic (76% and 49%, respectively) versus normal pregnancies (25% and 21%, respectively). The increased occurrence of positive p21 expression in the hypoxic group was highly significant (p<0.001). Similarly, analysis for hif1α expression indicated a significantly higher frequency of positive expression in the hypoxic group (p<0.001). There was no significant difference between the hypoxic and the normal groups regarding VEGF, mdm2 and ERCC5 genes.

**Table 1:** Prevalence of mRNA expressions in normal and hypoxic pregnancies.

		Normal	Hypoxic	
Gene	Expression	(n = 72) n (%)	(n = 41) n (%)	χ^2^ (p value)
p21	−	54 (75%)	10 (24%)	
	+	18 (25%)	31 (76%)	<0.001
Hif1α	−	57 (79%)	21 (51%)	
	+	15 (21%)	20 (49%)	<0.001
VEGF	−	55 (76%)	26 (63%)	
	+	17 (24%)	15 (37%)	NS (0.21)
Mdm2	−	47 (65%)	19 (46%)	
	+	25 (35%)	22 (54%)	NS (0.16)
ERCC5	−	17 (23%)	6 (15%)	
	+	55 (77%)	35 (85%)	NS (0.46)

The number of cases (n) and percentage of cases (%) in each group with no expression (−) and positive expression (+). χ^2^ tests are shown, significant results (p<0.05). NS, not significant.

A correlation between hif1α expression and p21 expression was evident, as indicated in the joint distribution table (**Table 2**). In order to determine whether the expression of p21 and hif1α are independently associated with hypoxic cases, a multivariate logistic regression was performed. A significant model has emerged with a highly significant effect for p21 (p<0.001) and a borderline significant effect for hif1α (p = 0.055), implying that, given the information on p21, there is not much additional information from determining hif1α.

**Table 2:** Joint distribution of p21 and hif1α in the study population.

	Hif1α (−)	Hif1α (+)
**p21 (−)**	57	7
**p21 (+)**	25	24

No expression (−), Positive (+).

### An association between currently used parameters for detection of complicated pregnancies and the different genes expression

Next, the relationships between the different genes (expressing versus not expressing) and fetal and maternal clinical characteristics (**Table S1**) were analyzed. Among the common parameters used to detect preeclampsia or complicated pregnancies are maternal blood hypertension, presence of proteinuria, abnormal fetoplacental blood flow, measured by fetoplacental Doppler and IUGR. Logistic regression analysis was used on the full data to investigate whether distributions of the various categorical variables differ from samples expressing or not expressing the different genes in the maternal plasma. Results, depicted in **Table 3**, indicate significant association between hypertension and hif1α (p<0.04), VEGF (p<0.03) and ERCC5 (p<0.02) expression in the maternal plasma. Urine protein, abnormal fetoplacental Doppler and IUGR did not reach significant values among all genes tested. For outcomes that did not meet the ANCOVA assumptions (normal distribution and constant error variance), p-values were determined by the bootstrap (**Table 4**). No significant differences were observed for additional parameters after adjustments of the results for the hypoxia and normal status.

**Table 3:** Association between hypertension, proteinuria, abnormal fetoplacental Doppler and IUGR with the expressions of the different genes in the maternal plasma.

	p values			
Gene	Hypertension	Proteinuria	Abnormal Doppler	IUGR
**p21**	0.461	0.636	0.318	0.218
**Hif1α**	**0.04**	0.929	0.894	0.926
**VEGF**	**0.034**	0.797	0.614	0.258
**ERCC5**	**0.024** *	0.307	0.098	0.43
**Mdm2**	0.845	0.468	1	0.292

For p21, hif1α, VEGF and mdm2, logistic regression statistic was used.

* For ERCC, Fisher's test was used. Significant results (p<0.05) in bold.

**Table 4:** Relation of clinical data to genes expression in the presence or absence of hypoxia.

		Normal		Hypoxia		p value
Age	Gene	−	+	−	+	
	**p21**	33.57	34.22	31.4	32.77	0.8
	**Hif1α**	33.66	34.18	31.4	33.45	0.26
	**VEGF**	33.58	34.23	31.52	33.93	0.2
	**ERCC5**	34.12	33.62	33.5	31.97	0.51
	**Mdm2**	34.34	32.6	32.05	32.35	0.32
**Birth week**	**Gene**	38.25	38.07	33.67	34.12	0.84^a^
	**p21**	38.27	37.84	34.11	33.91	0.59^a^
	**Hif1α**	38.15	38.39	34.8	32.55	0.11^a^
	**VEGF**	38.19	38.21	35.48	33.68	0.2^a^
	**ERCC5**	38.38	37.88	34.53	33.4	0.15^a^
	**Mdm2**	38.25	38.07	33.67	34.12	0.84^a^
**Fetal centile**	**Gene**	61.68	49.49	5.6	5.74	0.1^a^
	**p21**	60.92	47.64	5.29	6.15	0.21^a^
	**Hif1α**	59.22	57.5	5.27	6.47	0.91^a^
	**VEGF**	63.35	56.74	4.67	5.88	0.23^a^
	**ERCC5**	59.54	57.46	4.65	6.75	0.98^a^
	**Mdm2**	61.68	49.49	5.6	5.74	0.1^a^
**Birth centile**	**Gene**	58.31	47.78	4.6	6.81	0.3^a^
	**p21**	57.21	47.18	7.29	5.2	0.21^a^
	**Hif1α**	55.91	54.94	5.96	6.8	0.95^a^
	**VEGF**	63.76	53.18	4.17	6.74	0.28^a^
	**ERCC5**	56.32	54.48	5.75	6.95	0.94^a^
	**Mdm2**	58.31	47.78	4.6	6.81	0.3^a^
**Hemoglobin**	**Gene**	11.45	11.81	11.42	12.02	0.118
	**p21**	11.48	11.85	11.99	11.7	0.885
	**Hif1α**	11.62	11.26	11.86	11.87	0.472
	**VEGF**	11.78	11.44	11.75	11.82	0.416
	**ERCC5**	11.65	11.31	11.92	11.69	0.243
	**Mdm2**	11.45	11.81	11.42	12.02	0.118

Average of each parameter in the study groups is shown. p-values determined by the bootstrap are marked by the letter a.

### Gene expression in the maternal plasma of twin pregnancies

The study group included 19 twin pregnancies which were analyzed separately. There were 10 hypoxic twin pregnancies and 9 normal pregnancies. The prevalence of gene expressions in normal twin pregnancies versus complicated twin pregnancies, as defined upon sample collection, were analyzed due to the small sample sizes by Fisher's exact test. As indicated in **Table 5**, although positive p21 expression was much more common in the hypoxic twin pregnancies, nevertheless this difference did not reach statistical significance, most probably due to the small sample size. No significant differences between the groups were observed with the expression of the additional genes examined.

**Table 5:** Gene's expression in the maternal plasma of twin pregnancies.

	Normal		Hypoxia		
Gene	−	+	−	+	p value
**p21**	6	3	2	8	0.07
**Hif1α**	0	9	3	7	0.21
**VEGF**	1	8	7	3	0.58
**ERCC5**	2	7	3	7	1
**Mdm2**	4	5	7	3	0.37

Fisher's exact test for the expression of the different genes in hypoxia versus normal twin pregnancies. No expression (−), positive expression (+).

## Discussion

Tissue hypoxia during pregnancy, particularly preeclampsia, may affect both the mother and the fetus. Several modalities to diagnose hypoxia related disorders during pregnancy include biochemical markers such as PIGF [35], [36], sFLT1 [35], Endoglin [37] and PP13 [38]. When combined with maternal history, mean blood pressure and uterine artery Doppler, a detection rate of approximately 90% with 10% false positive cases is achieved [39]. As current modalities are not sufficient, more reliable methods for noninvasive preeclampsia monitoring are under research.

In recent years, plasma cell free RNA has been detected in various conditions and identification of possible markers, including for hypoxia during pregnancy are sought [40]. Several laboratories reported on an association between fetal growth and circulating RNA in the maternal plasma [41], [42], [43]. Hypoxia-inducible factors (HIFs) [6] and p53 [25], [26], are key transcriptional regulators of the hypoxic response. Both hif1α and p53 were shown to be regulated in placentas of pregnancies complicated by fetal growth retardation [27], [44], [45], [46]. However, to the best of our knowledge, all studies to date regarding hif1α and p53 were performed on placental tissues from mouse models or human subjects. We hypothesized that in complicated pregnancies, up regulation of hif1α and p53 pathways will lead to the accumulation of downstream cell free target genes in the maternal circulation and may serve as noninvasive diagnosis. This prediction was supported by our demonstration of an association between increased incidence of circulating p21 expression, a direct p53 target, as well as hif1α with complicated pregnancies. In accordance with our results, work done in placental tissues indicated that while mdm2 expression was not significant, a correlation with p21 was also documented [45]. They concluded that this association is reminiscent of that following exposure to hypoxia, highlighting the correlation of our results with data from placental tissues and their reliability as a possible tool for noninvasive diagnosis. Among the genes which did not discriminate between complicated and normal pregnancies in our work as well as others [47] was VEGF, the main ligand for the VEGF receptor (VEGFR). These results were unexpected as a significant difference between the expression of circulating VEGFR in preeclampsia versus controls was reported [43], [47].

The discovery of the presence of placental-derived fetal RNA in maternal plasma has opened up exciting opportunities for noninvasive prenatal diagnosis [28], [48], not limited by the fetal gender or genotype. However, maternal serum biomarkers investigated to date for the evaluation of fetal growth had suboptimal diagnostic sensitivity and predictive power [49], [50]. In our work, a strong association between p21 and hif1α expression patterns and IUGR was documented, indicating that p21, with or without hif1α expression, might be a good marker for hypoxic pregnancies and growth retardation. Finally, as with singleton pregnancies, p21 expression is much more common in hypoxic twin pregnancies than in normal twin pregnancies, even though the cohort size was too small for a statistically significant result.

To conclude, we present here data suggesting that p21 and hif1α mRNA plasma expressions may be used as markers for complicated pregnancies. More work is needed to increase the assay's sensitivity for future clinical use.

## Supporting Information

Table S1 **Characteristics of the study cohort:** H, Hypoxia; N, Normal; NA, Not available; G, Number of pregnancies; P, Previous pregnancies; HTN, Hypertension; *, Smoker; **, Twins; ***, Dollberg 2005.
(PDF)

Table S2 **Selected genes induced by (A) hypoxia and (B) p53.**
(PDF)

Table S3 **Delta Ct and Delta-Delta Ct calculations for the study cohort.**
(PDF)

Table S4 **Analysis of the study population:** Significant results (p<0.05) are highlighted by a grey background. Average (Avg), standard deviation (SD), interquartile range (IQR), hypoxia (H), normal (N).
(JPG)
