# Supplementary material for: Cell Free Expression of hif1α and p21 in Maternal Peripheral Blood as a Marker for Preeclampsia and Fetal Growth Restriction
Source: PLoS One. 2012 May 16;7(5):e37273. doi: 10.1371/journal.pone.0037273 (PMC3353943; doi:10.1371/journal.pone.0037273)
Supplement: Table S1 — Characteristics of the study cohort: H, Hypoxia; N, Normal; NA, Not available; G, Number of pregnancies; P, Previous pregnancies; HTN, Hypertension; *, Smoker; **, Twins; ***, Dollberg 2005. (PDF) [file pone.0037273.s001.pdf]

## Table S1: Characteristics of the study cohort

| | | | Estimated fetal weight | | | Actuall birth weight | | Apgar | | Maternal data | | | | | | | | Genes | | | | |
|---|---|---|---|---|---|---|---|---|---|---|---|---|---|---|---|---|---|---|---|---|---|---|
| Pt# | H/N | Sample | | Centiles | Birth | | Centiles | | | Age | HB | MCV | Intact flows | P | G | Urine protein | HTN | p21 | hif1a | vegf | mdm2 | ercc5 |
| | | week | (g) | *** | week | (g) | *** | 1 | 5 | years | | | Yes/No | # | # | Yes/No | Yes/No | | | | | |
| 1 | H | 23 | 300 | 3 | 25.71 | 360 | 3 | 4 | 5 | 26 | 13 | 78 | No | 3 | 7 | No | Yes | + | + | - | + | + |
| 2 | N | 24 | 650 | 29 | 37.43 | 2395 | 10 | 9 | 10 | 42 | 11 | 86.9 | NA | 0 | 4 | No | No | + | + | - | - | - |
| 3 | N | 38 | 2700 | 31 | 37.43 | 2390 | 10 | 9 | 10 | 40 | 14 | 89.7 | NA | 2 | 4 | No | No | + | - | + | - | + |
| 4 | N | 27 | 875 | 23 | 39.57 | 2950 | 24 | 9 | 10 | 29 | 11 | 91.7 | NA | 2 | 3 | No | No | - | + | - | - | + |
| 5 | H | 34 | 1609 | 5 | 35.00 | 1865 | 6 | 10 | 10 | 33 | 12 | 86.4 | Yes | 0 | 1 | No | No | + | - | - | - | + |
| 6 | H | 36 | 1867 | 6 | 36.29 | 1457 | 3 | 4 | 9 | 41 | 14 | 97 | No | 5 | 6 | No | Yes | + | + | + | NA | NA |
| 7 | H | 36 | 2160 | 9 | 36.71 | 2230 | 12 | 9 | 10 | 29 | 13 | 93.2 | No | 0 | 1 | No | No | + | + | - | + | + |
| 8 | H | 34 | 1212 | 3 | 35.14 | 1145 | 3 | 9 | 10 | 40 | 12 | 80.4 | No | 2 | 3 | Yes | Yes | + | + | - | + | + |
| 9 | N | 38 | 3850 | 95 | 38.29 | 4215 | 97 | 9 | 10 | 41 | 11 | 90.2 | NA | 2 | 7 | No | No | + | + | + | + | + |
| 10 | N | 38 | 2690 | 10 | 38.57 | 2690 | 17 | 9 | 10 | 30 | 12 | 87 | NA | 1 | 2 | No | No | + | + | - | - | + |
| 11 | N | 36 | 2400 | 22 | 37.29 | 2835 | 43 | 9 | 10 | 26 | 11 | 80.8 | Yes | 0 | 1 | No | No | + | + | + | - | + |
| 12* | H | 34 | 1433 | 3 | 35.29 | 1785 | 4 | 9 | 10 | 37 | 11 | 89.6 | No | 0 | 1 | No | Yes | + | + | - | - | + |
| 13** | H | 33 | 1700/1377 | 20/6 | 33.86 | 1790/1390 | 23/6 | 8/8 | 9/9 | 30 | 12 | 86.2 | No | 0 | 1 | No | No | + | - | + | - | + |
| 14** | H | 31 | 1259/1079 | 14/5 | 35.29 | 2210/1570 | 23/3 | 9/9 | 10/10 | 38 | NA | NA | No | 0 | 2 | No | No | + | + | + | - | - |
| 15 | H | 35 | 1971 | 9 | 36.14 | 2065 | 6 | 9 | 10 | 40 | NA | NA | Yes | 0 | 1 | No | Yes | + | + | + | + | + |
| 16 | H | 29 | 893 | 8 | 31.29 | 1015 | 3 | 9 | 10 | 36 | NA | NA | No | 0 | 2 | No | No | - | + | + | + | + |
| 17 | H | 24 | 405 | 3 | 24.43 | 371 | 3 | 0 | 0 | 25 | 14 | 93 | No | 0 | 1 | Yes | Yes | + | + | + | + | + |
| 18 | N | 38 | 3860 | 94 | 38.71 | 3620 | 87 | 9 | 10 | 31 | 12 | 89 | NA | 0 | 1 | No | No | + | - | + | - | + |
| 19 | H | 37 | 2259 | 6 | 37.71 | 1935 | 57 | 9 | 10 | 36 | NA | NA | Yes | 0 | 1 | No | No | + | + | + | - | - |
| 20 | N | 37 | 2900 | 50 | 37.43 | 2990 | 57 | 8 | 9 | 44 | 11 | NA | NA | 1 | 2 | No | No | - | - | + | - | + |
| 21 | N | 39 | 3400 | 93 | 39.29 | 4065 | 96 | 9 | 10 | 36 | 13 | 85.7 | NA | 6 | 7 | No | No | - | - | - | - | + |
| 22 | N | 38 | 3700 | 90 | 38.14 | 3700 | 90 | 9 | 10 | 36 | 12 | 83.5 | NA | 1 | 2 | No | No | + | + | + | - | + |
| 23** | H | 33 | 2020/1450 | 65/9 | 32.00 | 1925/1355 | 76/11 | 9/9 | 10/10 | 29 | 11 | 82.3 | No | 1 | 2 | No | No | + | + | + | - | + |
| 24** | H | 29 | 1352/736 | 68/3 | 33.29 | 2380/1210 | 93/3 | 9/9 | 10/10 | 24 | 13 | 95.2 | No | 1 | 2 | No | No | + | - | - | - | - |
| 25** | H | 35 | 1349/1342 | 5/5 | 32.14 | 1670/1355 | 43/11 | 9/9 | 10/10 | 30 | 11 | 90.7 | No | 0 | 1 | Yes | No | + | - | - | - | - |
| 26 | N | 37 | 3280 | 79 | 37.57 | 3060 | 63 | 9 | 10 | 36 | 12 | NA | NA | 3 | 4 | No | No | - | - | - | - | + |
| 27 | N | 38 | 2940 | 53 | 37.86 | 3360 | 82 | 9 | 10 | 24 | 10 | NA | NA | 2 | 3 | No | No | + | - | - | - | - |
| 28 | N | 37 | 3200 | 75 | 37.57 | 2965 | 55 | 9 | 10 | 37 | 12 | NA | NA | 2 | 3 | No | No | - | - | - | - | - |
| 29 | N | 39 | 3900 | 92 | 39.71 | 3720 | 85 | 9 | 10 | 32 | 12 | 87.3 | NA | 0 | 1 | No | No | - | - | - | - | - |
| 30 | N | 39 | 3980 | 94 | 39.43 | 4290 | 97 | 10 | 10 | 38 | 11 | 88.4 | NA | 2 | 3 | No | No | - | - | - | - | - |
| 31 | N | 38 | 3085 | 49 | 38.29 | 3085 | 49 | 9 | 10 | 30 | 11 | 86.3 | NA | 1 | 2 | No | No | - | - | - | - | - |
| 32 | N | 38 | 3165 | 65 | 38.57 | 3165 | 65 | 9 | 10 | 37 | 11 | NA | NA | 1 | 4 | No | No | - | - | + | - | - |
| 33 | N | 38 | 3120 | 52 | 38.86 | 3120 | 52 | 10 | 10 | 28 | 10 | 78.9 | NA | 1 | 2 | No | No | - | - | - | - | - |
| 34 | N | 38 | 2700 | 18 | 38.43 | 2700 | 18 | 9 | 10 | 32 | 12 | NA | NA | 2 | 3 | No | No | + | - | + | - | + |
| 35** | N | 37 | 3500/2800 | 97/77 | 38.00 | 3275/2505 | 95/36 | 7/9 | 9/10 | 32 | 11 | 92 | NA | 0 | 1 | No | No | - | - | - | + | + |
| 36* | N | 38 | 3700 | 90 | 38.57 | 3935 | 97 | 9 | 10 | 38 | 12 | NA | NA | 1 | 6 | No | No | - | - | + | - | - |

| | | | Estimated fetal weight | | | Actuall birth weight | | Apgar | | Maternal data | | | | | | | | Genes | | | | |
|---|---|---|---|---|---|---|---|---|---|---|---|---|---|---|---|---|---|---|---|---|---|---|
| Pt# | H/N | Sample | | Centiles | Birth | | Centiles | | | Age | HB | MCV | Intact flows | P | G | Urine protein | HTN | p21 | hif1a | vegf | mdm2 | ercc5 |
| | | week | (g) | *** | week | (g) | *** | 1 | 5 | years | | | Yes/No | # | # | Yes/No | Yes/No | | | | | |
| 37 | N | 38 | 3000 | 58 | 38.43 | 3505 | 81 | 9 | 10 | 36 | 12 | NA | Yes | 2 | 4 | No | No | + | - | - | - | - |
| 38 | H | 31 | 973 | 3 | 32.29 | 1055 | 3 | 7 | 9 | 34 | 12 | 97.5 | No | 0 | 1 | Yes | Yes | + | - | + | - | + |
| 39 | H | 30 | 743 | 3 | 34.43 | 1090 | 3 | 9 | 9 | 23 | 12 | 85.6 | No | 2 | 4 | No | No | + | - | - | - | - |
| 40 | H | 34 | 1529 | 3 | 36.29 | 2115 | 7 | 9 | 10 | 26 | NA | NA | Yes | 0 | 1 | No | No | + | - | - | - | + |
| 41 | H | 35 | 1985 | 4 | 36.57 | 1990 | 4 | 9 | 10 | 33 | NA | NA | No | 1 | 3 | No | No | + | + | - | + | + |
| 42** | H | 30 | 1222/757 | 23/3 | 31.00 | 1210/865 | 10/3 | 8/7 | 9/9 | 25 | NA | NA | No | 0 | 4 | No | No | + | + | - | + | + |
| 43 | H | 25 | 586 | 5 | 27.86 | 670 | 4 | 4 | 8 | 29 | NA | NA | No | 0 | 1 | Yes | Yes | + | + | + | + | + |
| 44* | N | 37 | 2700 | 31 | 37.86 | 2635 | 25 | 9 | 10 | 28 | NA | NA | Yes | 2 | 5 | No | No | - | - | - | - | + |
| 45* | N | 38 | 2750 | 21 | 38.29 | 2750 | 21 | 9 | 10 | 42 | NA | NA | NA | 1 | 5 | No | No | - | - | - | + | + |
| 46** | N | 37 | 2745/2700 | 73/69 | 37.43 | 3115/2180 | 94/19 | 9/9 | 10/10 | 32 | NA | NA | NA | 0 | 1 | No | No | + | - | - | - | - |
| 47 | N | 38 | 2805 | 24 | 38.14 | 2805 | 24 | 9 | 10 | 26 | NA | NA | NA | 2 | 5 | No | No | + | + | - | + | + |
| 48 | N | 39 | 3030 | 31 | 39.14 | 3030 | 31 | 9 | 10 | 32 | NA | NA | NA | 1 | 2 | No | No | - | - | + | - | + |
| 49 | N | 38 | 3500 | 81 | 38.86 | 3440 | 78 | 7 | 8 | 35 | NA | NA | NA | 1 | 3 | No | Yes | + | - | - | - | + |
| 50 | N | 38 | 3550 | 83 | 38.29 | 3275 | 66 | 9 | 10 | 32 | NA | NA | NA | 0 | 1 | No | No | - | - | - | - | + |
| 51 | N | 38 | 3600 | 86 | 38.43 | 3370 | 74 | 9 | 10 | 23 | NA | NA | NA | 1 | 2 | No | No | - | - | - | + | + |
| 52 | N | 38 | 2760 | 21 | 38.57 | 2440 | 6 | 9 | 10 | 31 | NA | NA | Yes | 0 | 1 | No | No | - | - | + | - | + |
| 53 | H | 38 | 2580 | 10 | 38.86 | 2980 | 40 | 9 | 10 | 35 | 11 | NA | NA | 1 | 2 | No | No | + | - | + | + | + |
| 54 | N | 38 | 3600 | 86 | 38.29 | 3845 | 94 | 9 | 10 | 37 | 14 | NA | NA | 1 | 2 | No | No | - | - | - | - | - |
| 55 | N | 38 | 3000 | 42 | 38.71 | 2805 | 25 | 9 | 10 | 35 | 11 | NA | NA | 1 | 2 | No | No | - | - | - | - | + |
| 56 | N | 38 | 4060 | 95 | 38.71 | 4190 | 97 | 9 | 10 | 31 | 10 | NA | Yes | 1 | 2 | No | Yes | - | - | - | - | + |
| 57 | N | 37 | 3400 | 84 | 37.29 | 2965 | 55 | 9 | 10 | 37 | 11 | NA | NA | 2 | 4 | No | No | - | - | - | - | + |
| 58 | N | 39 | 2746 | 52 | 39.14 | 3570 | 77 | 9 | 10 | 34 | 12 | NA | Yes | 0 | 1 | No | No | - | - | - | - | + |
| 59** | N | 36 | 2900/2900 | 65/65 | 36.71 | 2805/2475 | 87/59 | 9/9 | 10/10 | 35 | 11 | NA | NA | 0 | 1 | No | No | - | - | - | - | - |
| 60 | N | 39 | 3450 | 69 | 39.43 | 3400 | 64 | 9 | 9 | 31 | 12 | NA | NA | 1 | 2 | No | No | - | - | - | - | - |
| 61** | N | 38 | 2940/2800 | 86/40 | 38.29 | 2900/2730 | 75/58 | 9/9 | 10/10 | 31 | 12 | NA | NA | 0 | 1 | No | No | - | - | - | - | + |
| 62 | H | 26 | 717 | 14 | 30.57 | 1075 | 11 | 9 | 10 | 34 | 11 | 83.5 | Yes | 0 | 1 | Yes | Yes | + | + | + | + | + |
| 63 | N | 38 | 3200 | 59 | 39.00 | 3105 | 38 | 9 | 10 | 22 | 11 | 92.2 | NA | 1 | 2 | No | No | - | - | - | + | + |
| 64 | N | 38 | 2805 | 24 | 38.86 | 2805 | 24 | 9 | 10 | 39 | 12 | 91.3 | NA | 2 | 4 | No | No | - | - | - | + | + |
| 65 | N | 37 | 3400 | 84 | 37.86 | 3005 | 59 | 9 | 10 | 37 | 12 | NA | NA | 2 | 3 | No | No | - | - | - | - | - |
| 66 | N | 38 | 3065 | 48 | 38.29 | 3065 | 48 | 9 | 10 | 29 | 13 | 86.7 | NA | 0 | 1 | No | No | - | - | - | + | + |
| 67 | N | 38 | 3150 | 55 | 38.57 | 3150 | 55 | 9 | 10 | 27 | 12 | NA | NA | 0 | 3 | No | No | - | - | + | + | + |
| 68 | N | 38 | 3100 | 51 | 38.29 | 3350 | 72 | 9 | 10 | 33 | 9.7 | NA | Yes | 1 | 2 | No | No | - | - | + | + | + |
| 69 | N | 38 | 2963 | 38 | 38.29 | 3190 | 58 | 9 | 10 | 24 | 10 | 72.5 | NA | 1 | 2 | No | No | - | - | - | - | + |
| 70 | N | 39 | 3250 | 51 | 39.29 | 3035 | 31 | 9 | 10 | 40 | 12 | NA | NA | 1 | 2 | No | No | - | - | - | - | + |
| 71 | N | 38 | 3390 | 75 | 38.00 | 3390 | 75 | 9 | 10 | 31 | 13 | NA | NA | 9 | 11 | No | No | - | + | - | - | + |
| 72 | N | 38 | 3520 | 82 | 38.29 | 3520 | 82 | 9 | 10 | 37 | 13 | NA | NA | 9 | 11 | No | No | + | + | - | - | + |

| | | | Estimated fetal weight | | | Actuall birth weight | | Apgar | | Maternal data | | | | | | | | Genes | | | | |
|---|---|---|---|---|---|---|---|---|---|---|---|---|---|---|---|---|---|---|---|---|---|---|
| Pt# | H/N | Sample | | Centiles | Birth | | Centiles | | | Age | HB | MCV | Intact flows | P | G | Urine protein | HTN | p21 | hif1a | vegf | mdm2 | ercc5 |
| | | week | (g) | *** | week | (g) | *** | 1 | 5 | years | | | Yes/No | # | # | Yes/No | Yes/No | | | | | |
| 73 | N | 37 | 2605 | 23 | 37.57 | 2605 | 23 | 9 | 10 | 37 | 14 | NA | NA | 4 | 5 | No | No | - | + | - | - | + |
| 74* | N | 38 | 3195 | 59 | 38.29 | 3195 | 59 | 9 | 10 | 41 | 12 | NA | NA | 0 | 2 | No | No | - | - | - | - | - |
| 75 | H | 29 | 793 | 4 | 31.00 | 865 | 3 | 9 | 10 | 28 | 13 | 94.8 | No | 0 | 2 | No | Yes | - | + | + | + | + |
| 76 | H | 37 | 2005 | 5 | 39.00 | 2795 | 14 | 9 | 10 | 31 | 14 | 86.5 | Yes | 2 | 3 | No | No | + | + | - | - | + |
| 77 | H | 24 | 349 | 3 | NA | 320 | 3 | 0 | 0 | 29 | 12 | 87.1 | No | 0 | 1 | No | Yes | + | - | + | - | + |
| 78 | H | 34 | 1856 | 6 | 34.86 | 1865 | 5 | 7 | 8 | 28 | 11 | 86.7 | No | 0 | 1 | No | No | + | + | - | - | + |
| 79 | H | 36 | 1989 | 4 | 36.43 | 2360 | 20 | 9 | 10 | 37 | 13 | 96.1 | Yes | 2 | 4 | No | No | + | - | - | - | + |
| 80 | N | 38 | 2800 | 40 | 38.14 | 3040 | 45 | 9 | 10 | 31 | 13 | 92.7 | NA | 1 | 3 | No | No | + | - | - | - | + |
| 81 | N | 41 | 3745 | 77 | 41.29 | 3745 | 77 | 9 | 10 | 35 | 12 | NA | NA | 1 | 3 | No | No | - | - | - | + | + |
| 82 | N | 38 | 2755 | 21 | 38.57 | 2755 | 21 | 9 | 10 | 46 | 11 | NA | NA | 0 | 2 | No | No | + | - | - | - | + |
| 83 | H | 26 | 527 | 3 | 32.14 | 720 | 3 | 8 | 9 | 41 | 12 | 84.7 | No | 1 | 3 | Yes | Yes | + | + | - | - | + |
| 84 | H | 35 | 1458 | 3 | 36.00 | 1460 | 3 | 9 | 10 | NA | 12 | 95 | No | 0 | 1 | No | No | + | - | - | - | + |
| 85 | H | 35 | 1657 | 3 | 35.57 | 1519 | 3 | 9 | 10 | 29 | 12 | 91.7 | Yes | 0 | 1 | Yes | No | - | - | - | + | + |
| 86 | H | 36 | 1900 | 3 | 36.29 | 1870 | 3 | 9 | 10 | 33 | 13 | 82.9 | No | 6 | 8 | Yes | Yes | - | - | - | - | + |
| 87 | H | 37 | 2159 | 9 | 37.14 | 2100 | 3 | 9 | 10 | 35 | 11 | 94.5 | Yes | 1 | 2 | No | No | + | + | - | + | + |
| 88 | H | 30 | 1514 | 3 | 36.00 | 1695 | 3 | 9 | 9 | 31 | 9.9 | 87.7 | Yes | 0 | 2 | No | No | - | + | - | - | + |
| 89 | H | 29 | 615 | 3 | 31.29 | 835 | 3 | 10 | 10 | 22 | 13 | 93.1 | No | 0 | 8 | No | No | + | - | - | + | + |
| 90** | H | 35 | 2581/1720 | 83/8 | 37.00 | 3056/1920 | 92/6 | 9/9 | 10/10 | 25 | 11 | 95 | No | 0 | 3 | No | No | + | - | - | + | + |
| 91 | H | 26 | 415 | 3 | 28.29 | 510 | 3 | 9 | 10 | 35 | 11 | 87 | No | 0 | 2 | No | Yes | + | - | + | + | + |
| 92 | H | 26 | 540 | 3 | 29.57 | 600 | 3 | 5 | 9 | 40 | 12 | 99 | No | 0 | 1 | No | Yes | + | - | + | + | + |
| 93 | H | 38 | 2039 | 3 | 38.43 | 2015 | 3 | 9 | 10 | 30 | 14 | 94 | No | 0 | 2 | No | No | + | - | - | + | + |
| 94 | N | 38 | 3370 | 74 | 38.00 | 3910 | 95 | 9 | 10 | 36 | 12 | 87 | NA | 2 | 3 | No | No | - | - | - | + | + |
| 95 | N | 38 | NA | NA | 38.14 | 2325 | 3 | 9 | 10 | 40 | 11 | NA | NA | 2 | 3 | No | No | - | - | - | - | + |
| 96 | N | 39 | 3820 | 90 | 39.00 | 3505 | 73 | 9 | 10 | 38 | 11 | NA | NA | 1 | 2 | No | No | - | - | - | - | + |
| 97 | N | 38 | 3670 | 89 | 38.43 | 3705 | 90 | 9 | 10 | 32 | 11 | 90 | NA | 1 | 2 | No | No | - | - | - | - | + |
| 98 | N | 40 | 3850 | 87 | 41.71 | 3925 | 87 | 9 | 10 | 31 | 14 | 89 | NA | 3 | 5 | No | No | - | - | - | + | - |
| 99 | N | 38 | 3200 | 59 | 38.00 | 3265 | 65 | 9 | 10 | 30 | 11 | 85 | NA | 2 | 5 | No | No | - | - | - | + | + |
| 100 | H | 34 | 2311 | 17 | 36.86 | 2215 | 11 | 9 | 10 | 34 | 8.7 | 87 | Yes | 1 | 2 | No | Yes | - | + | + | + | + |
| 101 | N | 38 | 3200 | 59 | 40.43 | 3175 | 36 | 9 | 10 | 38 | 11 | 91 | NA | 2 | 3 | NA | NA | - | - | + | + | + |
| 102** | N | 36 | 2700/2445 | 80/56 | 37.14 | 2918/2582 | 57/51 | 9/9 | 10/10 | 36 | 12 | 87 | Yes | 0 | 2 | Yes | Yes | + | - | - | - | + |
| 103 | N | 31 | 2000 | 79 | 38.57 | 3280 | 66 | 9 | 10 | 35 | 9.5 | 85 | NA | 1 | 2 | No | No | - | - | - | - | + |
| 104 | H | 29 | 630 | 3 | 34.57 | 1076 | 3 | 9 | 10 | 33 | 14 | 90 | Yes | 0 | 1 | No | No | - | - | + | - | - |
| 105 | N | 31 | 1366 | 45 | 37.00 | 2464 | 15 | 9 | 10 | 33 | 13 | 90 | Yes | 0 | 1 | No | No | - | - | - | - | - |
| 106 | N | 35 | 2200 | 41 | 37.86 | 3146 | 71 | 9 | 10 | 34 | 10 | 85 | Yes | 4 | 5 | NA | NA | - | - | - | - | + |
| 107 | N | 38 | 4480 | 97 | 38.86 | 4500 | 97 | 9 | 10 | 31 | 8 | 66 | NA | 1 | 2 | No | No | - | - | + | + | + |
| 108** | N | 24 | 720/600 | 63/21 | 37.00 | 2684/2082 | 30/12 | 9/9 | 10/10 | 31 | 10 | 95 | Yes | 0 | 1 | No | No | - | - | - | + | + |

| | | | Estimated fetal weight | | | Actuall birth weight | | Apgar | | Maternal data | | | | | | | | Genes | | | | |
|---|---|---|---|---|---|---|---|---|---|---|---|---|---|---|---|---|---|---|---|---|---|---|
| Pt# | H/N | Sample | | Centiles | Birth | | Centiles | | | Age | HB | MCV | Intact flows | P | G | Urine protein | HTN | p21 | hif1a | vegf | mdm2 | ercc5 |
| | | week | (g) | *** | week | (g) | *** | 1 | 5 | years | | | Yes/No | # | # | Yes/No | Yes/No | | | | | |
| 109 | N | 29 | 1124 | 24 | 32.00 | 1696 | 33 | 8 | 10 | 28 | 11 | 96 | Yes | 1 | 2 | No | No | - | - | - | + | - |
| 110 | N | 38 | 3706 | 90 | 38.43 | 3706 | 90 | 9 | 10 | 39 | 13 | 87 | NA | 3 | 6 | No | No | - | - | - | + | + |
| 111 | N | 39 | NA | NA | 39.29 | 2664 | 8 | 9 | 10 | 34 | NA | NA | NA | 1 | 2 | No | No | + | - | + | + | + |
| 112 | H | 35 | 1950 | 9 | 37.14 | 2405 | 11 | 9 | 10 | 27 | 12 | 85 | Yes | 0 | 1 | No | No | - | - | - | + | + |
| 113 | H | 37 | 2388 | 10 | 37.71 | 2388 | 10 | 9 | 10 | 35 | NA | NA | Yes | 1 | 2 | No | No | + | - | - | - | - |
| 114** | N | 34 | 2057/1806 | 49/22 | 36.71 | 2515/2230 | 64/33 | 9/9 | 10/10 | 34 | 12 | 92 | Yes | 1 | 2 | Yes | Yes | + | - | + | + | + |
| 115 | N | 39 | 3622 | 80 | 39.29 | 3622 | 80 | 10 | 10 | 33 | NA | NA | NA | 1 | 2 | No | No | - | - | - | - | + |
| 116** | H | 28 | 950/910 | 10/7 | 33.86 | 1505/1484 | 10/11 | 8/9 | 9/10 | 43 | 12 | 96 | No | 1 | 2 | No | Yes | + | - | - | - | + |
| 117 | N | 25 | 660 | 43 | 25.29 | 696 | 21 | 6 | 10 | 31 | 10 | 90.4 | Yes | 1 | 2 | NA | NA | - | - | - | + | + |
| 118** | N | 26 | 850/730 | 44/17 | 30.43 | 1796/1298 | 93/34 | 3/8 | 8/10 | 31 | 10 | 91 | Yes | 0 | 2 | No | No | - | - | - | + | + |
| 119 | N | 36 | 2720 | 50 | 38.43 | 2920 | 33 | 9 | 10 | 28 | 12 | 81 | NA | 5 | 6 | No | No | + | - | - | + | + |
| 120 | H | 33 | 1300 | 3 | 37.43 | 2092 | 3 | 9 | 10 | 35 | 9.5 | 85 | Yes | 5 | 6 | No | No | + | + | - | + | - |
| 121** | N | 36 | 2290/2100 | 27/14 | 38.14 | 2276/2218 | 18/14 | 9/9 | 10/10 | 27 | 12 | 93 | NA | 1 | 2 | NA | No | - | - | - | + | + |
| 122** | H | 26 | 810/500 | 59/3 | 30.14 | 1463/672 | 60/3 | 9/8 | 10/9 | 34 | 10 | 89 | No | 0 | 1 | No | No | - | - | - | + | + |
| 123 | H | 32 | 1494 | 17 | 33.14 | 1624 | 13 | 7 | 8 | 31 | 12 | 102 | No | 0 | 1 | No | Yes | + | - | - | - | + |
| 124 | N | 34 | 2305 | 51 | 35.00 | 2338 | 34 | 9 | 10 | 41 | 11 | 97 | NA | 0 | 2 | No | No | + | + | - | + | + |
| 125 | H | 32 | 1450 | 14 | 33.00 | 1491 | 7 | 9 | 10 | 33 | 12 | 82 | Yes | 6 | 9 | Yes | Yes | + | - | - | + | + |
| 126 | N | 34 | 2300 | 51 | 36.57 | 2900 | 65 | 9 | 10 | 31 | 11 | 87 | Yes | 3 | 12 | No | No | - | - | + | + | + |
| 127 | H | 26 | 502 | 3 | 27.00 | 438 | 3 | 6 | 9 | 24 | 9.1 | 94 | No | 0 | 4 | Yes | Yes | - | - | - | - | + |
| 128 | H | 27 | 540 | 3 | 31.00 | 794 | 3 | 5 | 9 | 39 | 11 | 93.6 | NA | 0 | 1 | No | No | - | - | - | - | - |
| 129** | H | 32 | 1782/853 | 57/3 | 34.43 | 2424/836 | 85/3 | 9/9 | 10/9 | 28 | 12 | 87 | No | 1 | 3 | No | No | - | - | - | - | + |
| 130 | N | 39 | 3400 | 64 | 39.00 | 3424 | 66 | 9 | 10 | 32 | 11 | NA | NA | 0 | 1 | No | No | - | - | - | + | + |
| 131 | N | 39 | 3088 | 36 | 39.29 | 3088 | 36 | 9 | 10 | 28 | 10 | NA | NA | 1 | 2 | No | No | - | - | - | + | + |
| 132 | N | 39 | 3425 | 66 | 39.29 | 3425 | 66 | 9 | 10 | 40 | 12 | NA | NA | 2 | 4 | No | No | - | - | - | + | + |

| | |
|---|---|
| H | Hypoxia |
| N | Normal |
| NA | Not available |
| G | Number of pregnancies |
| P | Previous deliveries |
| HTN | Hypertension |
| * | Smoker |
| ** | Twins |
| *** | Dollberg 2005 |
