# Supplementary material for: Cell Free Expression of hif1α and p21 in Maternal Peripheral Blood as a Marker for Preeclampsia and Fetal Growth Restriction
Source: PLoS One. 2012 May 16;7(5):e37273. doi: 10.1371/journal.pone.0037273 (PMC3353943; doi:10.1371/journal.pone.0037273)
Supplement: Table S2 — Selected genes induced by (A) hypoxia and (B) p53. (PDF) [file pone.0037273.s002.pdf]

Table S2: Selected genes induced by (A) hypoxia and (B) p53

A

	Gene symbol	Accession #	Assay #
1	Hif1α	Nm_001530	Hs00936372_m1
2	VEGF	Nm_001025366	Hs99999070_m1
3	TGFβ3	Nm003239	Hs00234245_m1

B

	Gene symbol	Accession #	Assay #	Fold increase by p53
1	TP53	Nm_000546	Hs01034249_m1	-
2	P21 (CDKN1A)	Nm_000389	Hs01121168_m1	21
3	ERCC5	Nm_000123.2	Hs01557031_m1	58
4	MDM2	Nm_006878	Hs00234753_m1	14
5	TP53I3 (PIG3)	Nm_004881	Hs00153280_m1	11
6	NOTCH1	Nm_017617	Hs00413187_m1	26
7	PIGF	Nm_002643	Hs00601696_m1	15
8	BTG2	Nm_006763	Hs00198887_m1	10
9	ZMAT3 (WIG1)	Nm_022470	Hs01074692_m1	24
10	APAF1	Nm_013229	Hs00185508_m1	7
11	FAS	Nm_152873	Hs00910107_m1	54
12	ANGPTL2	Nm_012098	Hs00765775_m1	37
13	PUMA (BBC3)	Nm_014417	Hs00248075_m1	30
14	IGFBP6	Nm_002178	Hs00181853_m1	30
15	GDF15	Nm_004864	Hs00171132_m1	32
