# Supplementary material for: Cell Free Expression of hif1α and p21 in Maternal Peripheral Blood as a Marker for Preeclampsia and Fetal Growth Restriction
Source: PLoS One. 2012 May 16;7(5):e37273. doi: 10.1371/journal.pone.0037273 (PMC3353943; doi:10.1371/journal.pone.0037273)
Supplement: Table S3 — Delta Ct and Delta-Delta Ct calculations for the study cohort. (PDF) [file pone.0037273.s003.pdf]

Table S3: Delta Ct and Delta-Delta Ct calculations for the study cohort

		A					B				
		Delta Ct					Delta-Delta Ct from calibrator				
Pt#	H/N	p21	hif1a	vegf	mdm2	ercc5	p21	hif1a	vegf	mdm2	ercc5
1	H	7.1	9.1	-	10.9	10.6	64.9	0.27	-	0.07	0.09
2	N	6.9	10.2	-	-	-	74.6	0.13	-	-	-
3	N	12.6	-	10.3	-	11	1.43	-	0.06	-	0.07
4	N	-	11.7	-	-	11.1	-	0.04	-	-	0.07
5	H	13.6	-	-	-	13	0.72	-	-	-	0.02
6	H	11.8	9.6	10.9	-	-	2.5	0.19	0.04	-	-
7	H	9.4	10.3	-	9.1	8.8	13.2	0.12	-	0.23	0.33
8	H	15.4	7.9	-	12.4	14.4	0.21	0.62	-	0.02	0.01
9	N	15.3	10.7	16.6	14.4	12.3	0.22	0.09	0	0.01	0.03
10	N	11.9	12.2	-	-	10.8	2.33	0.03	-	-	0.08
11	N	9.5	11.2	9.4	-	11	12.3	0.06	0.1	-	0.07
12*	H	12.5	12.3	-	-	12.6	1.54	0.03	-	-	0.02
13**	H	9.8	-	8.1	-	11	9.99	-	0.25	-	0.07
14**	H	9.1	12.4	10.1	-	-	16.2	0.03	0.06	-	-
15	H	10.2	10.9	8.4	8.3	10	7.57	0.08	0.21	0.40	0.14
16	H	-	10.3	10.5	11.6	10.7	-	0.12	0.05	0.04	0.09
17	H	11.9	10	13.1	11.2	10.7	2.33	0.15	0.01	0.05	0.09
18	N	15.2	-	12.3	-	11	0.24	-	0.01	-	0.07
19	H	11.3	8.1	12.9	8.4	-	3.53	0.54	0.01	0.37	-
20	N	-	-	12.6	-	11.2	-	-	0.01	-	0.06
21	N	-	-	-	-	11.5	-	-	-	-	0.05
22	N	11.3	10	11.3	-	10.8	3.53	0.15	0.03	-	0.08
23**	H	11.1	7.5	12.1	-	10.6	4.06	0.82	0.02	-	0.09
24**	H	10.2	-	-	-	-	7.57	-	-	-	-
25**	H	8.4	-	-	-	-	26.4	-	-	-	-
26	N	-	-	-	-	9.3	-	-	-	-	0.23
27	N	11.6	-	-	-	-	2.87	-	-	-	-
28	N	-	-	-	-	-	-	-	-	-	-
29	N	-	-	-	-	-	-	-	-	-	-
30	N	-	-	-	-	-	-	-	-	-	-
31	N	-	-	-	-	-	-	-	-	-	-
32	N	-	-	9.1	-	-	-	-	0.13	-	-
33	N	-	-	-	-	-	-	-	-	-	-
34	N	11.8	-	13	-	11.9	2.5	-	0.01	-	0.04
35**	N	-	-	-	10.4	9.9	-	-	-	0.09	0.15
36*	N	-	-	10.1	-	-	-	-	0.06	-	-
37	N	10	-	-	-	-	8.7	-	-	-	-
38	H	9.5	-	10.7	-	-	12.3	-	0.04	-	-
39	H	9.8	-	-	-	-	9.99	-	-	-	-
40	H	11.7	-	-	-	11	2.68	-	-	-	0.07
41	H	7.8	8.7	-	12.3	9.3	40	0.36	-	0.02	0.23
42**	H	11.7	10.8	-	12.2	10.8	2.68	0.08	-	0.03	0.08
43	H	14.5	8.7	11.2	12.3	7.7	0.38	0.36	0.03	0.02	0.71
44*	N	-	-	-	-	9.1	-	-	-	-	0.27
45*	N	-	-	-	-	7.8	-	-	-	-	0.66
46**	N	-	-	-	-	13	-	-	-	-	1.0875
47	N	9.8	10.5	-	9.5	10.4	9.99	0.1	-	0.17	0.11
48	N	-	-	13.3	-	9.8	-	-	0.01	-	0.16
51	N	-	-	-	9.7	8.5	-	-	-	0.15	0.41
52	N	-	-	9.3	-	10.6	-	-	0.11	-	0.09
53	H	11	-	-	9	10.3	4.35	-	-	0.25	0.12
54	N	-	-	-	-	-	-	-	-	-	-
55	N	-	-	-	-	4.2	-	-	-	-	7.98
56	N	-	-	-	-	11.3	-	-	-	-	0.06

		Delta Ct					Delta-Delta Ct from calibrator				
Pt#	H/N	p21	hif1a	vegf	mdm2	ercc5	p21	hif1a	vegf	mdm2	ercc5
57	N	-	-	-	-	8.7	-	-	-	-	0.35
58	N	-	-	-	-	11.5	-	-	-	-	0.05
59**	N	-	-	-	-	-	-	-	-	-	-
60	N	-	-	-	-	-	-	-	-	-	-
61**	N	-	-	-	-	10.4	-	-	-	-	0.11
62	H	12	10.4	13.1	10.9	12.6	2.17	0.11	0.01	0.07	0.02
63	N	-	-	-	12.1	10.3	-	-	-	0.03	0.12
64	N	-	-	-	9.1	10.6	-	-	-	0.23	0.09
65	N	-	-	-	9.1	11.4	-	-	-	0.23	0.05
66	N				10.5	14	-	-	-	0.09	0.01
68	N	-	-	12.9	7.8	9.5	-	-	0.01	0.57	0.20
69	N					9.4	-	-	-	-	0.2172
70	N	-	-	-	-	10.6	-	-	-	-	0.09
71	N	-	-	-	-	11.5	-	-	-	-	0.05
72	N	-	-	-	-	8.4	-	-	-	-	0.43
73	N	-	6.9	-	-	5.6	-	1.25	-	-	3.03
74*	N	-	-	-	-	9.4	-	-	-	-	0.22
75	H	-	-	10.5	10.9	9.3	-	-	0.05	0.07	0.23
76	H	12.6	12.8	-	-	11.9	1.43	0.02	-	-	0.04
77	H	9.9	-	10.9	-	10.1	9.32	-	0.04	-	0.13
78	H	10.2	11.7	-	-	9.9	7.57	0.04	-	-	0.15
79	H	-	-	-	-	11.8	-	-	-	-	0.04
80	N	10.2	-	-	-	9.9	7.57	-	-	-	0.15
81	N	-	-	-	12.9	11.3	-	-	-	0.02	0.06
82	N	12.8	-	-	-	13.4	1.25	-	-	-	0.01
83	H	12.2	10	-	-	11.9	1.89	0.15	-	-	0.04
84	H	11.7	6.8	-	-	8.4	2.68	1.34	-	-	0.43
85	H	-	-	-	-	9.8	-	-	-	-	0.16
86	H	-	-	-	-	8.9	-	-	-	-	0.31
87	H	11.6	12.9	-	11.9	10.4	2.87	0.02	-	0.03296	0.11
88	H	-	13.5	-	-	11.8	-	0.01	-	-	0.04
89	H	10.6	-	-	-	8.6	5.74	-	-	-	0.38
90**	H	9.8	-	-	-	9.1	9.99	-	-	-	0.27
91	H	10.3	-	11.3	7.3	7.7	7.07	-	0.03	0.80	0.71
92	H	10.4	-	12.4	8	8.6	6.59	-	0.01	0.49	0.38
93	H	10.1	-	-	7.9	10.6	8.12	-	-	0.53	0.09
94	N	-	-	-	9.7	10.1	-	-	-	0.15	0.13
95	N	-	-	-	-	9.7	-	-	-	-	0.18
96	N	-	-	-	-	8.5	-	-	-	-	0.41
97	N	-	-	-	-	8	-	-	-	-	0.57
98	N	-	-	-	11.1		-	-	-	0.06	-
99	N	-	-	-	13.5	11.2	-	-	-	0.01	0.06
100	H		10.3	13.5	8.4	11.3	-	0.12	0.01	0.37294	0.0582
101	N	-	-	-	8.9	11.5	-	-	-	0.26	0.05
102**	N	11.5	-	-	-	11.1	3.08	-	-	-	0.07
103	N	-	-	-	-	10	-	-	-	-	0.14
104	H	-	-	8.1	-	-	-	-	0.25	-	-
105	N	-	-	-	-	-	-	-	-	-	-
106	N	-	-	-	-	7.1	-	-	-	-	1.07
107	N	-	-	10.1	7.6	8.1	-	-	0.06	0.65	0.53
108**	N	-	-	-	11.5	9.7	-	-	-	0.04	0.18
109	N	-	-	-	11.2		-	-	-	0.05	-
110	N	-	-	-	9.2	7.9	-	-	-	0.21	0.61
111	N	10.6	-	10.7	10.2	10.9	5.74	-	0.04	0.11	0.08
112	H	-	-	-	8.5	8.2	-	-	-	0.35	0.50
113	H	-1.5	-	-	-	-	10^3	-	-	-	-

		Delta Ct					Delta-Delta Ct from calibrator				
Pt#	H/N	p21	hif1a	vegf	mdm2	ercc5	p21	hif1a	vegf	mdm2	ercc5
114**	N	39.9	-	40.5	37.3	37.6	0	-	0	0.00	0.00
115	N	-	-	-	-	9.8	-	-	-	-	0.16
116**	H	9.3	-	-	-	9.1	14.1	-	-	-	0.27
117	N	-	-	-	9.1		-	-	-	0.23	-
118**	N	-	-	-	8.4	6.4	-	-	-	0.37	1.74
119	N	9.4	-	-	9.9	9.6	13.2	-	-	0.13	0.19
120	H	9.9	8.8	-	9.7	11.5	9.32	0.33	-	0.15	0.05
121**	N	-	-	-	12.7	9.9	-	-	-	0.02	0.15
122**	H	-	-	-	12	10.7	-	-	-	0.03	0.09
123	H	11.5	-	-	-	10.4	3.08	-	-	-	0.11
124	N	14.7	9.4	-	10.1	10.9	0.33	0.22	-	0.11	0.08
125	H	12.1	-	-	12.6	10.8	2.03	-	-	0.02	0.08
126	N	-	-	13.9	13.4	12.6	-	-	0	0.01	0.02
127	H	-	-	-	-	7.4	-	-	-	-	0.87
128	H	-	-	-	-	-	-	-	-	-	-
129**	H	-	-	-	-	9.3	-	-	-	-	0.23
130	N	-	-	-	8.7	7.7	-	-	-	0.30	0.71
131	N	-	-	-	10.8	7.8	-	-	-	0.07	0.66
132	N	-	-	-	10.5	8.7	-	-	-	0.09	0.35
k-562		13.1 ± 1.8	7.22 ± 1.2	6.127 ± 1.3	6.977 ± 1.2	7.197 ± 0.7	1	1	1	1	1
