# Supplementary figures and images for: Cell Free Expression of hif1α and p21 in Maternal Peripheral Blood as a Marker for Preeclampsia and Fetal Growth Restriction
Source: PLoS One. 2012 May 16;7(5):e37273. doi: 10.1371/journal.pone.0037273 (PMC3353943; doi:10.1371/journal.pone.0037273)

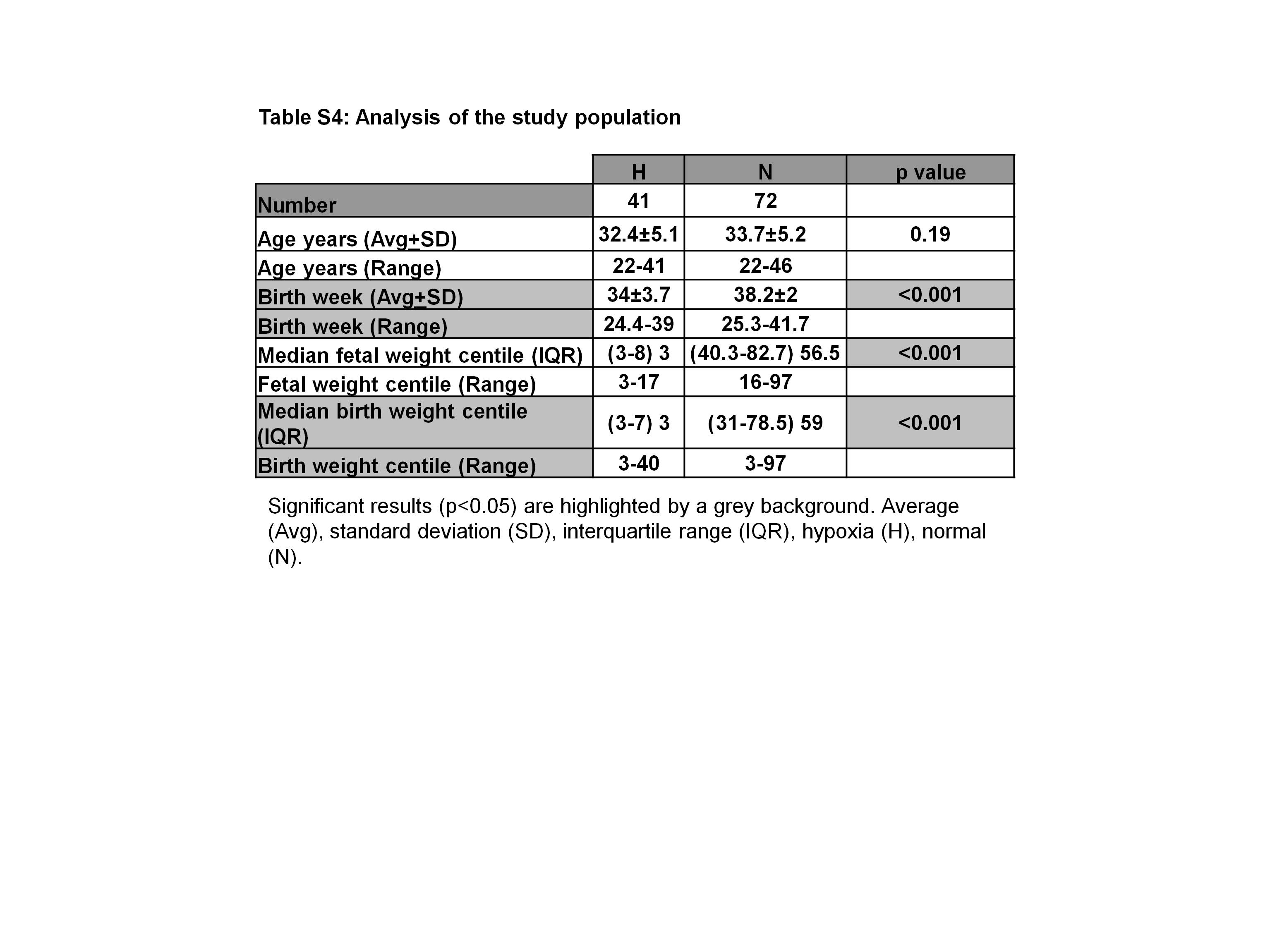

Supplement: Table S4 — Analysis of the study population: Significant results (p<0.05) are highlighted by a grey background. Average (Avg), standard deviation (SD), interquartile range (IQR), hypoxia (H), normal (N). (JPG) [file pone.0037273.s004.jpg]
